# Gene Expression Profiling of the Response to Interferon Beta in Epstein-Barr-Transformed and Primary B Cells of Patients with Multiple Sclerosis

**DOI:** 10.1371/journal.pone.0102331

**Published:** 2014-07-15

**Authors:** Rana Khsheibun, Tamar Paperna, Anat Volkowich, Izabella Lejbkowicz, Nili Avidan, Ariel Miller

**Affiliations:** 1 Rappaport Faculty of Medicine, Technion-Israel Institute of Technology, Haifa, Israel; 2 Division of Neuroimmunology and Multiple Sclerosis Center, Carmel Medical Center, Haifa, Israel; University of North Carolina at Greensboro, United States of America

## Abstract

The effects of interferon-beta (IFN-β), one of the key immunotherapies used in multiple sclerosis (MS), on peripheral blood leukocytes and T cells have been extensively studied. B cells are a less abundant leukocyte type, and accordingly less is known about the B cell-specific response to IFN-β. To identify gene expression changes and pathways induced by IFN-β in B cells, we studied the *in vitro* response of human Epstein Barr-transformed B cells (lymphoblast cell lines-LCLs), and validated our results in primary B cells. LCLs were derived from an MS patient repository. Whole genome expression analysis identified 115 genes that were more than two-fold differentially up-regulated following IFN-β exposure, with over 50 previously unrecognized as IFN-β response genes. Pathways analysis demonstrated that IFN-β affected LCLs in a similar manner to other cell types by activating known IFN-β canonical pathways. Additionally, IFN-β increased the expression of innate immune response genes, while down-regulating many B cell receptor pathway genes and genes involved in adaptive immune responses. Novel response genes identified herein, *NEXN*, *DDX60L*, *IGFBP4*, and *HAPLN3*, B cell receptor pathway genes, *CD79B* and *SYK*, and lymphocyte activation genes, *LAG3* and *IL27RA*, were validated as IFN-β response genes in primary B cells. In this study new IFN-β response genes were identified in B cells, with possible implications to B cell-specific functions. The study's results emphasize the applicability of LCLs for studies of human B cell drug response. The usage of LCLs from patient-based repositories may facilitate future studies of drug response in MS and other immune-mediated disorders with a B cell component.

## Introduction

Aberrant B cell activity is an underlying pathological mechanism in a range of immune-related disorders, from diseases that are primarily antibody mediated, such as systemic lupus erythematosus, to diseases considered to be primarily T cell driven, such as multiple sclerosis (MS) [1], [2]. Evidence for the role of B cells in MS includes the oligoclonal IgG bands present in the cerebrospinal fluid of most MS patients; the ectopic B cell follicles within germinal centers in meninges of MS patients; and the aberrant B cell cytokine response observed in MS patients [3]. The Epstein-Barr virus (EBV) that targets and infects B cells has been suggested as an MS risk factor, with recent supportive evidence including the increased disease severity in animals infected with the virus and the enrichment of EBV-infected B cells in MS lesions [4], [5]. The pivotal role of B cell in MS was further demonstrated following reports of the therapeutic efficacy of the B cell-depleting anti CD20 drug in MS clinical trials [6], [7]. Both antigen presenting functions of the B cell and antibody secretion contribute to disease pathogenesis [8], [9].

Whereas much attention has been allocated in recent years to investigate B cell functions in MS pathogenesis, specific information regarding the effects of the MS immunomodulatory drugs on B cell activity is still lacking. This is also true with respect to interferon beta (IFN-β), which is among the key first-line therapies approved for relapsing MS [10]. Gene expression analyses and studies of cellular signaling pathways activated in response to IFN-β mostly described the response in T cells, or peripheral blood leukocytes [11]–[13]. The IFN-β responses of small, yet important, cell populations such as B cells or monocytes have not received adequate attention. Indeed, accumulating evidence indicates immune cell-specific effects in gene expression are rife, including specifically for IFN-β, as shown by us and others for distinct genes and pathways in monocytes, in comparison to T cells [14]–[16].

Since B cells participate in both innate and adaptive immune functions, functions which are modulated by IFN-β [17] and contribute to MS pathogenesis, it is of interest to delineate the cellular pathways triggered by IFN-β in B cells of patients with MS. Previous studies described a role for IFN-β in B cell maturation [18], and in modulation of the TLR7 signaling pathway, a pathway involved in viral response and autoimmunity [19]–[21]. IFN-β was reported to increase transcription of the pro-survival genes encoding the B cell activating factor (*TNFSF13B/BAFF*), *STAT3*, and *STAT5* [15], [22], but also to suppress release of stimulatory cytokines such as IL-1β and IL23 [23]. In all, these studies focused on a few specific and known pathways, which suggest a complex manner in which IFN-β affects B cells. The discovery of other pathways, possibly B cell-specific, that are affected by IFN-β can benefit from a hypothesis-free approach, such as genome-wide gene expression analysis.

The study of primary B cell functions is hampered by their low percentage among peripheral blood leukocytes and their short lifespan in culture. However, human B cells can be transformed by the EBV to create lymphoblast cell lines (LCLs) that retain many B cell properties, including typical cell surface markers and immunoglobulin secretion [24], as well as phenotypic characteristics of the donor B cells [25]. The usage of LCLs carries important technical advantages since they are much more easily maintained compared to primary B cell cultures and the *in vitro* set up allows repeated and parallel experiments on the same samples. Moreover, recent studies have confirmed that LCLs preserve the inter-individual gene expression variability of primary B cells, including heritable patterns governed by genetic background [26], [27]. Despite the inherent limitations of research using transformed cell lines (reviewed in Welsh et al., 2009), it is increasingly recognized that LCLs derived from patient populations can be useful for studies of the effects of genetic variation on cellular function as related to disease and drug response [28]–[32]. Studies probing the regulatory functions of MS associated genomic regions have used genetic data from a LCL to demonstrate an overlap with B cell-specific transcriptionally active genomic regions [31], [32]. Specifically, LCLs may serve as a model to study genetic variance related to the drug effects with specific reference to B cell activity.

In this study, we used LCLs from people with MS to characterize the gene expression patterns of the response to *in vitro* IFN-β exposure. Whole genome analysis highlighted genes previously unknown to be affected by IFN-β. The results from analysis of LCLs, identifying new IFN-β response genes, were replicated in primary B cells, corroborating the relevance of the LCL system as a model for studying drug responses in B cells.

## Materials and Methods

### Study participants

This study was approved by the Carmel Medical Center Helsinki Committee and the Israeli Ministry of Health National Helsinki Committee for Genetics Studies. All participants provided a written informed consent. Participants (>18 years) were recruited at the MS center at Carmel Medical Center, Haifa. Participants included were patients with clinically definite or laboratory supported MS diagnosis according to Poser and MacDonald criteria, [33], [34] and healthy individuals as controls. Exclusion criteria for controls were presence of MS in family members up to third degree, or presence of any autoimmune or chronic inflammatory condition. Detailed demographic and clinical data were obtained from all subjects. Ethnicity was determined by the participant's self-report. MS disease type (relapsing-remitting, secondary-progressive, relapsing-progressive or primary-progressive) was recorded at date of phlebotomy. Blood samples were obtained from all participants, and peripheral blood mononuclear cells (PBMC) were purified using Ficoll gradient (NovaMed).

### Lymphoblastoid cell lines generation and culture conditions

Frozen PBMC samples from participants were EBV transformed at the European Collection of Cell Cultures, England (http://www.phe-culturecollections.org.uk/3083.aspx), and the National Laboratory for the Genetics of Israeli Populations at Tel Aviv University, Israel (http://nlgip.tau.ac.il). The LCLs were cultured in RPMI-1640 supplemented with 10% FBS, 2 mM L-glutamine, penicillin (100 U/ml), streptomycin (100 µg/ml), and nystatin (12.5 U/ml, Biological Industries). LCLs were assayed within one month of thawing for the different experimental assays to minimize the passage numbers and avoid immortalization and other changes associated with long term growth in culture [25], [35].

### Flow cytometry

LCLs were stained by multicolor flow cytometry with FITC anti-human CD19; APC-CY7 anti-human CD27; PerCP anti-human CD38; and APC anti-human CD24 (Biolegend). BD CompBeads (Becton and Dickinson) were used for compensation according to the manufacturer's instructions. Unstained cells were used to exclude background fluorescence and isotype controls to determine antibody specificity. LCL viability was monitored using 7-amino-actinomycin D (eBiosciences, USA). Data reading and acquisition was performed using the Flow Cytometer Cyan ADP (Beckman Coulter). FlowJo Software (TreeStar) was used for data analysis.

### Primary B cell Isolation

B cells were isolated from PBMCS using the negative selection Human B cell isolation kit (Miltenyi), or EasySep Human B cell enrichment kit (Stemcell) according to the manufacturers' instructions. All B cells preparations had at least 80% purity levels by CD19 flow cytometry.

### IFN-β incubation assay

Cells were incubated with 100 units/ml recombinant human IFN-β1a (InterferonSource) for 4, 16, or 48 hours. Cytokine concentrations used were within the range of reported serum IFN-β levels following drug injection in MS patients, and for the extended incubation times IFN-β was added to medium after 24 hours to account for cytokine half-life [36], [37]. Untreated cells were cultured in parallel and under identical culture conditions for comparison. LCLs and primary leukocyte IFN-β assays were performed at a density of 1.4 million cells/ml.

### RNA extraction

RNA from LCL cultures was extracted using the Roche RNA isolation kit. RNA from primary B cells was extracted using the Ambion RNAqueous® Micro Kit. The concentration of RNA was measured by NanoDrop ND-100 Spectrophotometer (NanoDrop Technologies). RNA samples processed for the microarray chip assays and all RNA samples derived from primary B cells were checked for quality using Experion (Bio-Rad).

### Microarray chip gene expression analysis

A total of 16 pairs of RNA samples from LCLs (IFN-β treated for 4 hours or untreated) were hybridized to Illumina BeadChip HumanHT-12 v4, according to the manufacturer's protocols. Raw data was processed by GenomeStudio software (Illumina) for quality control. Data were log2 transformed, and batch normalization was applied. Data were filtered for background noise and variance (variance ≥2^0.1^).

The gene expression dataset has been deposited in GEO with access no. GSE58240.

### Real Time RTPCR assays

cDNA synthesis was performed using M-MLV reverse transcriptase (Promega) with random hexamer primers (Biological Industries). Specific PCR primers for selected genes were designed by us or obtained from published primer databases (PrimerBank, http://pga.mgh.harvard.edu/primerbank/ or qPrimerDepot, http://primerdepot.nci.nih.gov), and were obtained from Sigma or Integrated DNA Technology, IDT (Table S1). RT-PCR analyses were performed using the FastStart Universal SYBR Green Master-Rox (Roche) on the AB 7300 sequence detection system in duplicates. The ubiquitin-conjugating enzyme E2 D2 (*UBE2D2*) gene was used as a reference gene as in previous and the present studies its levels were unaffected by IFN-β [38]. Relative quantification of mRNA expression was calculated using the comparative CT method and is shown as fold change of expression (2^−ΔΔ*C*^_T_) [39].

### Statistical Analysis

Statistical analysis of BeadChip gene expression array data was performed using JMP® Genomics version 6.0 software (SAS Institute Inc.). A total of 3035 probes out of the 47323 probes included in the BeadChip passed filtrations and were included in the statistical analysis. Principal component analysis and variance component analysis were performed as implemented in JMP Genomics. One-way ANOVA was performed to identify differentially expressed genes between IFN-β treated or untreated LCL samples, with correction for multiple testing by the False Discovery Rate (FDR) method [40]. Differentially expressed genes were defined as transcripts that had a log2(fold change of expression)≥1 and an FDR corrected P-value≤0.05. To select for novel genes that had not been previously recognized as IFN-β response genes, the differentially expressed gene list was tested against the Interferome v2.01 database, using the parameters of log2(fold change)>1, and for selected down regulated genes also log2(fold change)<−0.4 [41]. The IFN-β differentially expressed gene list was compared with the list of differentially expressed genes between LCLs and their counterpart B cells based on the data of Caliskan et al., with log2(fold change)>1 and FDR ≤0.05 [26].

Data from RT-PCR experiments and flow cytometry analyses of cell subset distributions were analyzed using SPSS version 18.0 (IBM), P-values ≤0.05 were considered significant, and Bonferroni correction was applied as relevant. To compare between data from LCLs groups or with primary B cells and PBMCs, Mann Whitney U test was used. For studying the IFN-β effect by comparing untreated samples to treated samples, the Wilcoxon signed rank test was applied.

### Pathway Analysis

Functional networks and canonical pathways affected by IFN-β treatment were explored with Ingenuity Pathways Analysis software (Ingenuity Systems). The data set containing the differentially expressed genes, their corresponding expression values, and P-values, was uploaded into Ingenuity software, and analysis was performed with a cutoff of P-value  = 0.05 and log2(fold change) = 0.4 to obtain the top canonical pathways and functions. Top functional networks significantly activated (Z score>2) were further explored, as were specific upstream and downstream connections for genes of interest for their B cell-related function.

## Results

### B cell-subsets composition across donor groups is similar

**Figure 1:**
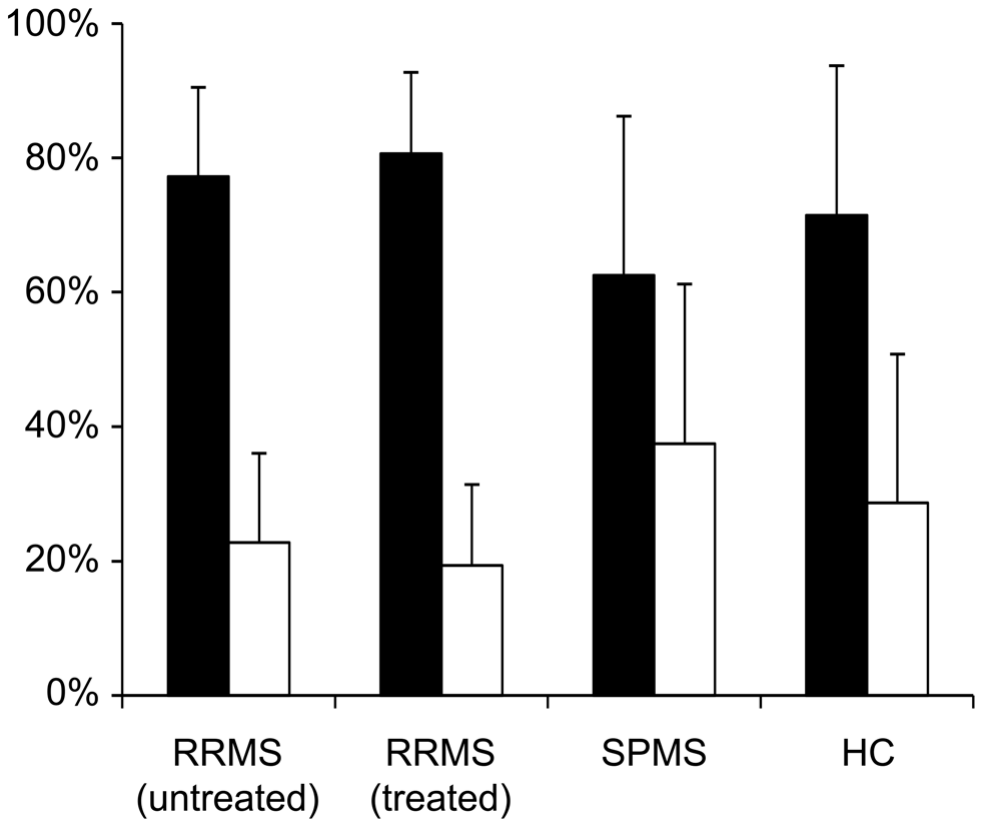
Flow cytometry indicates similarity in proportions of cells with a naïve B cell or memory B cell marker phenotype among LCLs from healthy donors and patients with MS. LCLs were typed for memory and naïve B cell cell-surface phenotype by flow cytometry, by CD19 and CD27 immunoreactivity. The percentage of cells for each subset is shown for LCLs from donors with relapsing remitting MS that did not receive any therapy prior to the time of phlebotomy (RRMS: untreated, n = 12), donors with relapsing remitting MS under IFN-β therapy (RRMS: treated, n = 12), donors with secondary progressive MS (SPMS, n = 9), and donors that were healthy controls (HC, n = 5). Black bars: CD19+CD27- naïve B cells; white bars: CD19+ CD27+ memory B cells. No differences were observed between all comparison groups (Mann Whitney test).

Both naïve B cells and memory B cells express the EBV receptor CD21 and hence are the major targets of the viral transformation [42]. Thus, overall variance in gene expression across LCL samples may be influenced by differences in B cell-subset composition between samples. To address this issue B cell surface markers CD19 and CD27 were analyzed in LCLs from healthy controls and MS donors, either treated with IFN-β or not at the time of phlebotomy, to evaluate B cell subset phenotype distributions [43] (Fig. 1). No differences were observed among the LCL groups in the major cell subset distributions representing naïve B cell-like and memory B cell-like LCLs. Analysis of variance for B cell subset distributions revealed low variance within independent tests of a specific sample, but a significant variance between LCLs from different donors (ANOVA, P≤0.001). These results indicate that the relative proportions of naïve B cell-like and memory B cell-like cell subsets remain stable in culture for each sample, at least within the short-term culture period used in the current study. Moreover, we did not detect any segregation in cell-surface marker distribution that was related to the donor's disease status or therapy status at the time of phlebotomy.

### Gene expression analysis of IFN-β response

**Figure 2:**
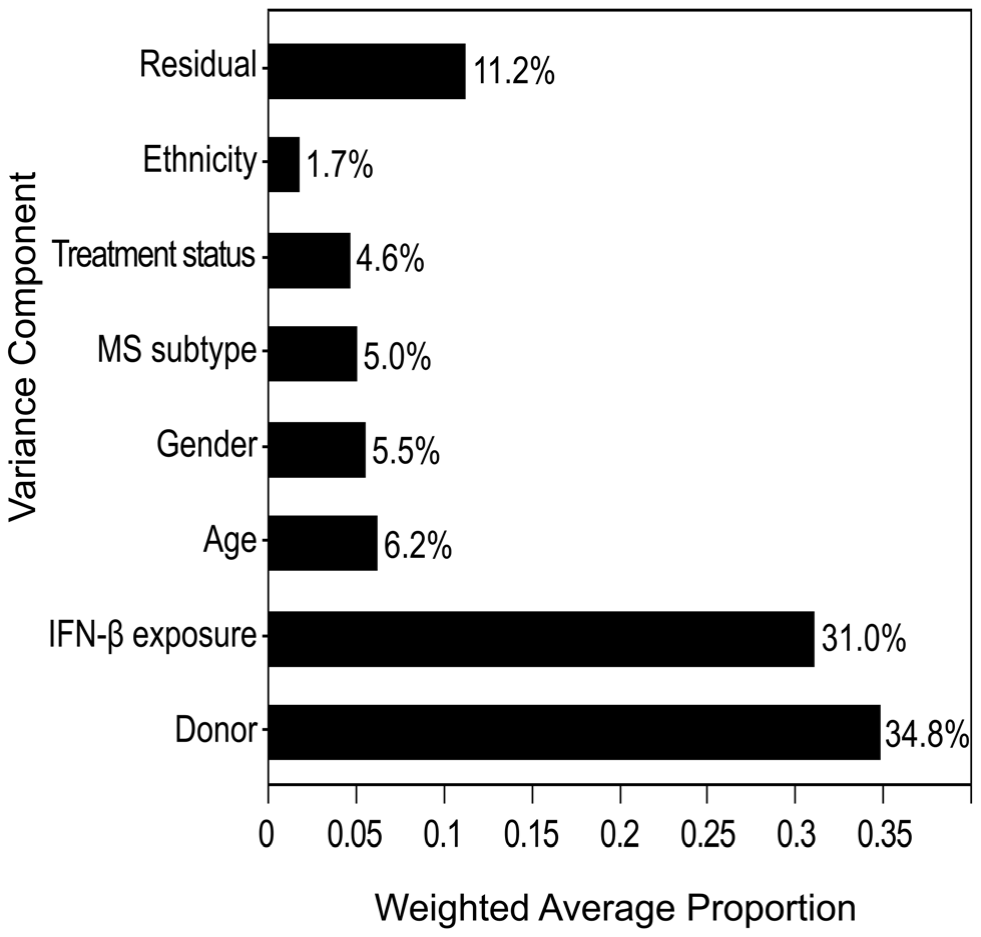
Proportion of variance in gene expression is explained by a major IFN-β response component and donor-specific components. Variance component analysis was modeled with the following components: Donor - representing variance contributed by unspecified differences between donors; IFN-β exposure (*in vitro* IFN-β treated LCL sample versus the untreated sample); Age (stratified to under 40 years old or above); Gender; MS subtype (relapsing remitting, relapsing progressive, secondary progressive), Treatment status (IFN-β treatment naïve donors, or donors treated for at least 1 year at the time of sample collection); and Ethnicity (Jewish/Arab). The Residual component models all the variability that cannot be attributed to any of the explicit variance components.

Whole genome gene expression analysis was performed for the *in vitro* response of 16 LCL samples to four hours IFN-β exposure. LCLs were obtained from donors affected with MS [63% females, Age median 38.5 yrs (range 20–59 yrs), of Jewish (50%) and Arab descent (50%), 81% with relapsing remitting MS, and 75% treated with IFN-β, at the time of blood collection]. Variance component analysis detected variance among donors and IFN-β response as major contributors to the overall variance in gene expression (Fig. 2). In addition, variance related to the donor state at time of phlebotomy, such as age, treatment status (whether donor was receiving IFN-β therapy or not), or disease subtype (relapsing remitting MS, secondary progressive MS or progressive relapsing MS) contributed each more than 5% to the overall variance.

**Figure 3:**
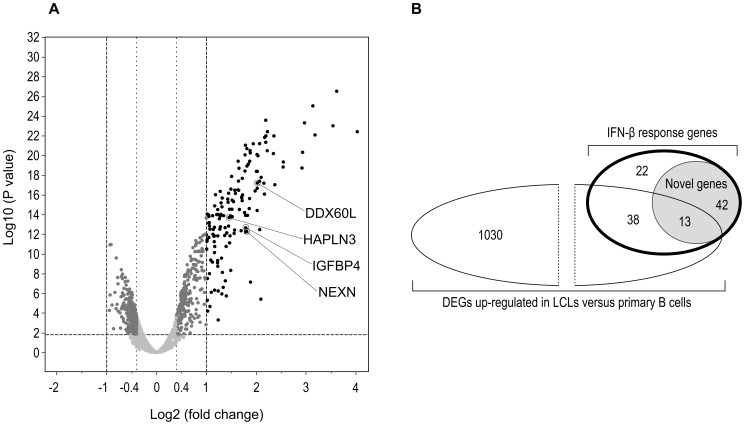
The LCL gene expression response to IFN-β. A: Volcano plot for the IFN-β gene expression response in LCLs. Log2(fold change) of expression levels following IFN-β exposure and P-values (one-way ANOVA) are depicted for each transcript probe. The horizontal dashed line represents the threshold for the adjusted P-value of 0.05. Vertical dashed line indicates threshold of log2(fold change)>1 and <−1. Differentially expressed genes (in black) are genes with log2(fold change) ≥1 and adjusted P-value ≤0.05, novel IFN-β response genes included in RTPCR validation analyses are marked. For pathways analyses a lower threshold was employed (dotted vertical line) with log2(fold change)≥0.4 (dark grey dots). B: Venn diagram describing the proportion of differentially expressed genes in response to IFN-β [log2(fold change)≥1, adjusted P-value<0.05, bold outlined ellipse] that are novel response genes (grey shade), and a comparison to the published differentially expressed genes up-regulated in LCLs in comparison to primary B cells, based on the data of Caliskan and colleagues [log2(fold change)≥1, adjusted P-value<0.05][26]. Definition of novel IFN-β response genes is based on the Interferome V2.01 database (accessed 19.8.13) search for the 115 IFN-β response differentially expressed genes. Numbers indicate the number of genes within each subgroup.

At our stringent cutoff, twofold change and above, at an adjusted P-value<0.05, 115 up-regulated genes were found following IFN-β treatment, but no down-regulated genes (Fig. 3A, Table S2 and Table S3). Cross-referencing the gene list with the Interferome V2.01 database [41] highlighted 55 genes that had not yet been reported as IFN-β response genes (Fig. 3B). Of these novel IFN-β response genes, 13 had been reported by Caliskan and colleagues as genes that are up-regulated in LCLs versus primary B cells and thus may be related to the viral transformation [26]. The remaining 42 genes may be considered as novel IFN-β response genes that are likely to be expressed in primary B cells as well, and from which four were selected for RTPCR validation as described next (3.3).

### Validation of novel IFN-β response genes in LCL and in primary B cells

Four novel IFN-β response genes, *NEXN*, *HAPLN3*, *DDX60L*, and *IGFBP4*, were selected for additional validation based on their robust fold change values on one hand, but lack of functional information with respect to B cell specific functions or connection to IFN-β related pathways. *NEXN* encodes nexilin, an F-actin binding protein proposed to function as a linker protein for the cytoskeleton and in focal adhesion junctions [44]. *HAPLN3* belongs to the hyaluronan and proteoglycan link protein family, and encodes a ubiquitous extracellular matrix protein [45]. The function of DDX60L can only be postulated from its homology to DDX60, an RNA helicase involved in the IFN antiviral response [46], [47]. The insulin-like growth factor binding protein 4, IGFBP4, is a modulator of the activity of the insulin growth factors [48].

**Figure 4:**
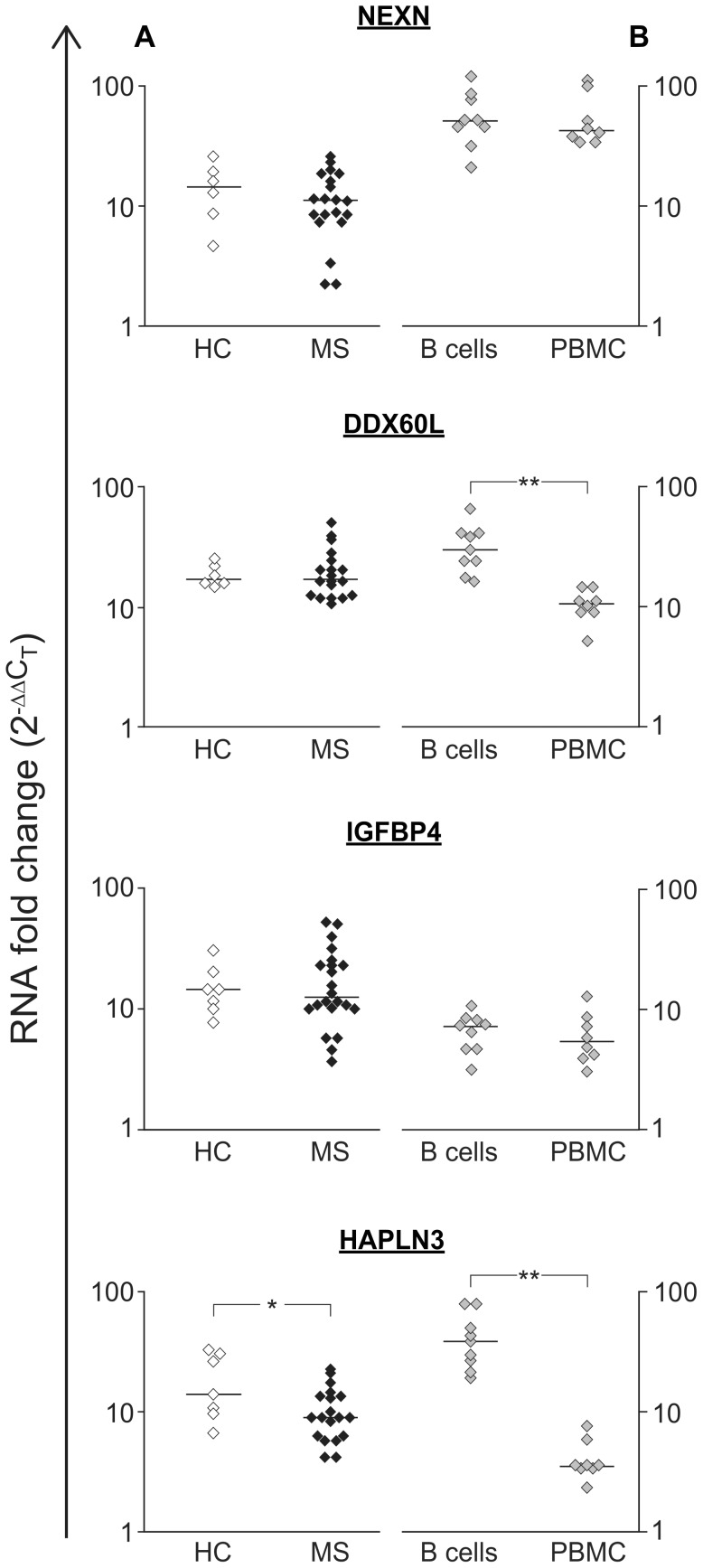
Validation of the gene expression response for four novel IFN-β response genes by real time RT-PCR. The change in expression levels (2-^ΔΔ*C*^_T_) following four hours of IFN-β exposure is shown for four genes that are novel IFN-β response genes. The horizontal bars represent the median values. A: Comparison of the IFN-β response in LCLs from healthy controls (HC, empty symbols, n = 7) and patients with multiple sclerosis (MS, full symbols, n = 20). Fold changes for IFN-β response were significant for both groups (P-value<0.007, Wilcoxon signed rank test). No difference was observed in the IFN-β response between controls and MS LCLs, except for *HAPLN3*, for which the difference was of a small magnitude (*P-value<0.05, Mann Whitney test). B: The IFN-β response in primary B cells (n = 9) and PBMC (n = 8) from healthy controls and MS patients. All genes displayed a significant response to IFN-β for both B cells and PBMC (P-value<0.001, Wilcoxon signed rank test). Significant differences between the response in B cells and PBMCs is indicated by ** P-value = 0.0003 (Mann Whitney test). The response magnitude of B cells and LCLs differed for all genes, however, these differences were small (P-value<0.03, Mann Whitney test).

Using real time RTPCR these four genes were validated as IFN-β response genes, first in LCLs from MS patients and healthy donors, followed by validation in primary B cells and PBMCs (Fig. 4). We did not detect differences in the response to IFN-β between LCLs from MS and healthy controls for *NEXN*, *DDX60L* and *IGFBP4*, but for *HAPLN3* a slight increase in expression was found in control samples (P = 0.048) (Fig. 4A). The expression of all four genes was significantly up-regulated (P≤0.01) following IFN-β exposure in B cells, although to a different extent than in LCLs (P<0.03, Fig. 4B). An IFN-β transcription response for the four genes was also observed in PBMCs; however, for *DDX60L* and *HAPLN3* the response was of a significantly lower magnitude than in B cells (P = 0.001), emphasizing the cell specific nature of this cytokine's response.

**Figure 5:**
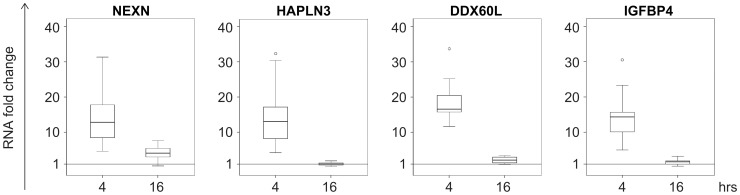
*NEXN*, *DDX60L*, *IGFBP4* and *HAPLN3* respond transiently to IFN-β. The expression levels of *NEXN*, *DDX60L*, *IGFBP4* and *HAPLN3* in LCLs were analysed by real time RT-PCR analysis after 4 or 16 hours of exposure to 100 u/ml IFN-β. Data is presented as a box plot of the 25^th^ and 75^th^ percentiles around the median values.

To study the temporal dynamics of IFN-β, we assessed its effect on transcription of the new IFN-β response genes at longer exposure times up to 48 hours. All four genes selected for validation appeared to have a transient response to IFN-β, suggestive of early response genes, as at 16 hours of exposure their expression returned to baseline levels (Fig. 5).

### IFN-β induces activation of canonical IFN pathways and affects other immune pathways in LCLs

**Figure 6:**
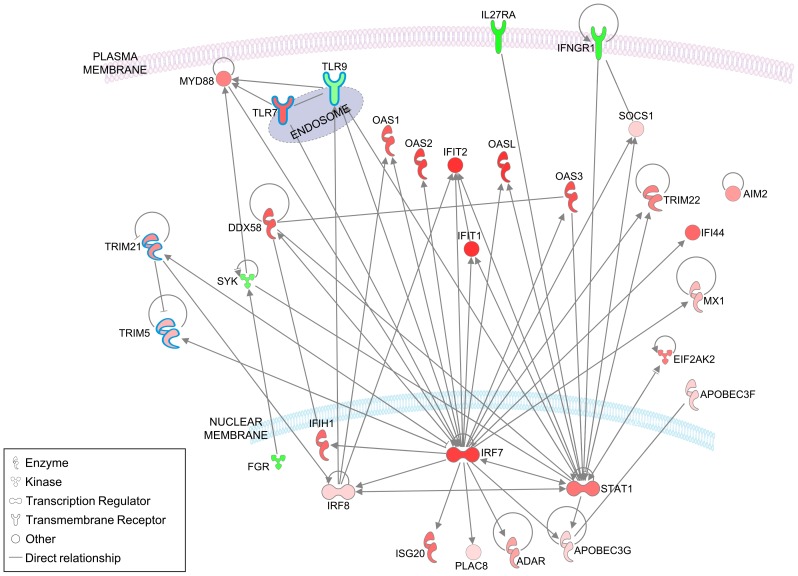
IFN-β activates the antimicrobial pathway and related innate immunity genes in LCLs. The pathway depicted includes the 27 genes of the of the 'antimicrobial response' pathway that showed increased activation following IFN-β exposure of LCLs (activation score Z = 2.779) by Ingenuity analysis. In addition, genes outlined in blue were manually added to the pathway based on their known participation in the innate response in B cells or other leukocytes, and include *TLR7* and *TLR9* [20] [74], *TRIM21* and *TRIM5* [75]–[77]. Only direct connections are featured, and subcellular location of gene products is as suggested by Ingenuity knowledge base. In green- down-regulated genes; in red- up-regulated genes, color intensity is proportional to the fold change in expression levels following IFN-β exposure.

Pathways analysis is especially useful for detection of coordinated expression changes of functionally related genes that individually do not pass the significance threshold. Ingenuity pathways analysis highlighted the known IFN response pathways, including the 'IFN signaling' pathway (P = 2.6E-13) and 'activation of IRF by cytosolic pattern recognition receptors' (P = 1.3E-6). In addition, the 'antimicrobial response' function is activated following IFN-β exposure (Z score = 2.8), shown in Figure 6, with the addition of nodes known to participate in the B cell innate response. The overall increased expression observed in this pathway is consistent with an IFN-β-induced activation of innate pathways in the LCLs. Thus, with respect to IFN-β canonical pathways, LCLs respond to IFN-β in a manner that is overall similar to other cell types.

**Figure 7:**
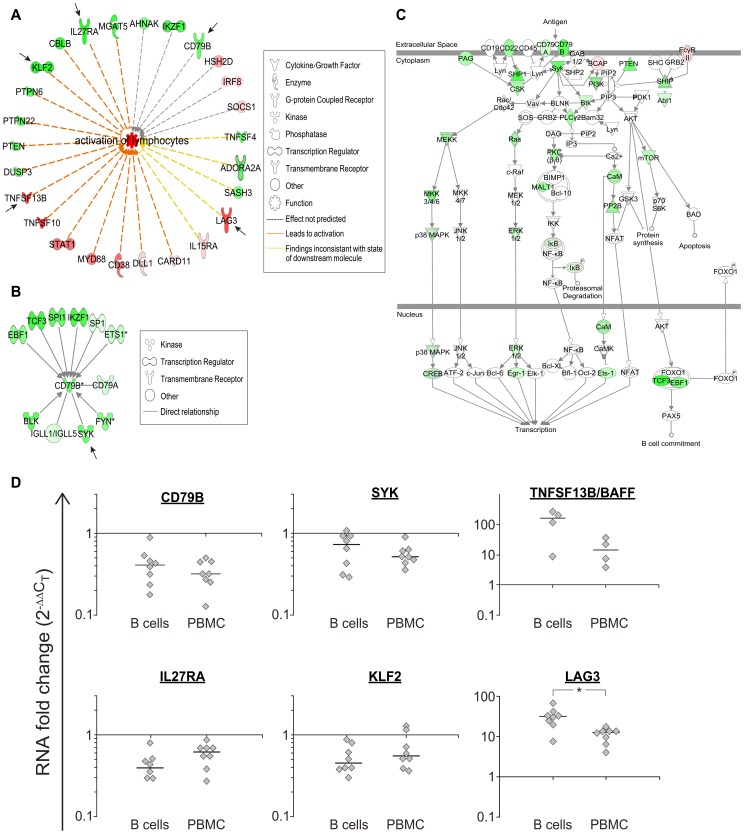
Networks of B cell-related genes are affected by IFN-β in LCLs. Network of genes included in the ‘Activation of Lymphocytes' function highlighted by Ingenuity analysis (Z = 2.33). Arrows points to IFN-β response genes that are not included in the Interferome database, and were included in a follow up validation analysis by quantitative RTPCR shown in D. In green- down-regulated genes; in red- up-regulated genes, color intensity is proportional to the fold change in expression levels following IFN-β exposure. A: An interaction network of CD79B, the immunoglobulin-β subunit of the B cell receptor signaling complex, with its regulators and interacting genes. The interacting nodes were limited to genes that were included in the filtered gene expression dataset, and were identified as *directly connected* to CD79B based on Ingenuity pathways analysis database. Notably, all the genes included in this network were found to be down-regulated by IFN-β (green) to some extent. Fold change and P-values are noted below each node. B: Decreased expression levels were observed for many of the genes included in the canonical B cell receptor signaling pathway in response to IFN-β. C: Quantitative reverse transcriptase RTPCR analysis of IFN-β response in primary B cells and PBMCs for genes marked in arrows in A and B. Genes were selected for validation because they were not identified by the Interferome database as IFN-β response genes, and are related to B cell function (*CD79B, SYK, TNFSF13B/BAFF*), or participate in the antimicrobial pathway depicted in Fig. 6 (*IL27RA*), or are related to activation of lymphocytes (*LAG3, KLF2*). * P-value = 0.007 (Mann Whitney test).

The top physiological systems affected were 'Hematological System Development and Function', and 'humoral immune response', both are systems that include functions of B cells (for both systems P<5.5E-03). Within the 'Hematological System Development and Function' category, the 'activation of lymphocytes' network was increased (Z scores >2), including classic B cell genes like *CD79B* and *TNFSF13B/BAFF*, and genes that are mainly known as T cell response regulators, like *LAG3* and *IL27RA* [49]–[52] (Fig. 7A). The prototype B cell molecule CD79B that is part of the B cell receptor (BCR) complex and is expressed from early B cell developmental stages [53] was significantly down-regulated in LCLs (adjusted P = 0.01), albeit at a small fold change (0.7). Interestingly, many of the direct interacting partners of CD79B, including upstream transcription factors were down-regulated as well (Fig. 7B). Moreover, other components of the BCR pathway also decreased in expression following IFN-β exposure, although the overall statistical significance did not reach the threshold (Fig. 7C).

Several of the genes included in the lymphocyte related networks highlighted by Ingenuity pathways analysis are currently not recognized as IFN-β response genes by the Interferome database, including *CD79B*, *SYK*, *IL27RA*, *LAG3*, and *KLF2*. Quantitative RTPCR on RNA from primary B cells and PBMCs exposed to IFN-β confirmed these genes as IFN-β response genes (Fig. 7D). We note that for *LAG3*, up-regulation of expression in B cells was threefold higher in comparison to PBMCs, emphasizing the cell specific nature of the IFN-β effect. *TNFSF13B/BAFF*, which is not yet included in the Interferome database as an IFN-β response gene, was also up-regulated in B cells, in agreement with previous reports [15], [22]. Thus, immune-related novel IFN-β response genes identified in LCLs were confirmed in primary B cells as IFN-β response genes.

## Discussion

In this study we report that the cytokine and immunomodulatory drug IFN-β activates prototype IFN canonical pathways in LCLs, including the antimicrobial innate immune response. Our novel findings comprise the decreased expression of several BCR pathway genes, including the immunoglobulin β component of the BCR CD79B together with its upstream regulators and downstream interacting proteins following exposure to IFN-β. Thus, the LCL response to a short IFN-β exposure overall is similar to the known lymphocyte response, but it also includes a response component of typical B cell genes and pathways. The effects of IFN-β on the BCR genes suggest a reduction of the capacity of the B cell to respond to antigen mediated signals, funneling its activity towards immediate innate system type of response. These results provide support to the applicability of LCLs as a model system to study the cellular effects of drug response, specifically with respect to B cell related functions.

We identified in this study over 50 genes that were not recognized previously as IFN-β response genes using LCLs. The majority of these novel response genes have similar expression levels in LCLs and primary B cells [26], and are therefore likely to be IFN-β response genes in B cells as well, as we were able to demonstrate for the genes *NEXN*, *HAPLN3*, *DDX60L*, and *IGFBP4*. Two of these genes, *HAPLN3* and *DDX60L*, displayed a large difference in the drug response between B cells and PBMCs, which may explain why they had not been previously identified as IFN-β response genes in studies using T cells or PBMCs. All four genes appear to be early response genes for IFN-β, and their transcription is under tight regulation, as indicated by the return to baseline levels after 16 hours of cytokine exposure. Presently, there is lack of functional data (as also indicated by lack of Ingenuity pathway connections) that can explain the contribution of the activity of these four genes to IFN-β-induced B cell activity. *NEXN* and *HAPLN3* may affect cell motility as cytoskeleton and extracellular matrix proteins, however, additional studies are required to assess their contribution to immune cell functional modulation by IFN-β. In the context of possible modes of action of IFN-β as an immunomodulatory therapy in MS, it is worth noting that IGFBPs have been suggested to be involved in regulation of remyelination and increased levels of IGFBP4 in MS lesions were reported [54]. Elevated levels of IGFBP4 were reported to inhibit proliferation and induce apoptosis of mice lymphocytes in lymphoid tissues [48], [55].

Some of the novel IFN-β response genes identified in this study, including *KLF2*, *LAG3* and *IL27RA*, are known as immune function genes; however they had not been studied in the context of IFN activity and the effect on B cells. KLF2, the Krüppel-like factor 2, is a transcription factor involved in B cell homeostasis, regulating the development and balance between the marginal zone and follicular B cell pools [56]. KLF2 transcriptionally regulates the expression of several cell surface receptors that are involved in cell motility, however it appears to have a differential effect between B and T cells [56]. In B cells, KLF2 was shown to affect plasma cell homing to the bone marrow, mainly via transcription of integrin β7. Thus, down-regulation of KLF2 by IFN-β in B cells may decrease the migration capacity of antibody secreting B cells, affecting the adaptive response arm of the B cell function. LAG3 is a CD4-like protein suggested to function as an inhibitory receptor to T cell activation and to regulate T cell homeostasis [50], [57]. LAG3 is suggested to exert its inhibitory actions on T cells by competitive binding to MHC class II. The large increase in LAG3 RNA levels mediated by IFN-β specifically in B cells, as shown herein, points to an inhibitory component on B cell activation that may be among the immunomodulatory effects of IFN-β. That the increase of LAG3 levels by IFN-β may serve to attenuate cell activation in both B and T cells is in line with the modulatory role attributed to IFN-β in MS, the augmentation of autoimmunity by LAG3 deficiency [58], and the increased activation of B cells occurring in MS [59]. IL27RA, a subunit of the receptor for IL27, is known to be required for maturation of B cells in the germinal centers through its signaling within the follicular T cells [51], it can induce IgG class switching in B cells [60], and is required for generation of high affinity anti-viral antibodies [52]. Thus, we suggest that down-regulation of IL27RA following IFN-β treatment may indicate the dampening of activation of B cell maturation, congruent with the effect of IFN-β on LAG3, decreased function of plasma cells due to KLF2 down-regulation, and the overall decreased expression of the B cell receptor pathway genes.

One of the important properties of the LCL as a research tool is that it can be used to study functional effects of genomic variation on expression, since LCLs recapitulate the donor variance in gene expression [26], although there is also contradictory evidence to this point [61], [62]. This property has been recently exploited in association studies of gene expression and disease, and with drug response [63]–[67]. In fact, Bolotin and colleagues demonstrate in their study the overall clustering of gene expression patterns of B cells and LCLs by donor [63]. Although our study was not designed to assess the effect of the donor's phenotype on the IFN-β response, our findings suggest that the gene expression variance among LCLs reflects, in addition to genetic variance, the donor's status at the time of phlebotomy, for characteristics such as age, disease state, and therapy status. The B cell-like subset composition of LCLs appears to be a sample-specific and reproducible characteristic that, similarly to gene expression patterns, may be related to the donor and his state at the time of phlebotomy. Studies employing animal models have been very instructive in deciphering immunological pathways, however, they have also revealed important differences in immune functions between mice and men, including B cell development and activity [68], [69]. Moreover, IFN type 1 is species-specific [70], [71], emphasizing the need to study the immunomodulatory properties of IFN-β activity in the context of human cells. Thus, analysis of drug response in experimental systems of animal models may fail to identify human-specific activity, and LCLs offer the advantage of an *in vitro* system that is based on human cells. Moreover, the study of complex drug interactions, including evaluation of drug mixtures, and comparative studies of drugs, can benefit from the *in vitro* experimental setting that allows controlled timing of drug application and dosage on one hand, while allowing consideration of the genetic and gene expression variance within the population, as expressed in the LCLs on the other hand.

There are several known and inherent caveats in the *in vitro* system of the LCLs, which call for awareness and caution in data interpretation: First, the effect of the viral transformation on the gene expression patterns, and that of the viral programmed transcription on cytokine and drug induced pathways [26], [63]. This is particularly pertinent when the study involves an antiviral cytokine or drug, like IFN-β. Secondly, the isolated and homogeneous cell culture *in vitro* lacks many of the functional interactions present in the *in vivo* multi-organ - multi-cellular environment, with regulatory feed-forward and feed-back effects emanating from the various cell types and their interactions. Thus, the LCL is an experimental system of lower complexity, with the advantages and disadvantages associated with a reductionist approach. Finally, time course of drug effects in the multicellular organism versus the cell culture is of an entirely different magnitude. The long term effects of many drugs are cumulative and can differ significantly from the short term effects, as recently demonstrated for IFN-β in MS [72]. With reference to the present study, our study design led to identification of many genes which displayed transient changes in expression levels and returned to baseline levels at longer IFN-β exposure time. Within the four hour time window of exposure to IFN-β the transcription response of both 'immediate-early' (expressed independently of protein translation) and 'secondary response genes' is expected [73]. The assessment of the temporal nature of the IFN-β response genes was beyond the scope of our study; however, extended time course analyses *in vitro*, that may be part of future studies, could contribute to the identification of sustained transcription patterns that may better resemble the functional effect of drugs *in vivo*.

## Conclusions

New IFN-β response genes were identified by focusing on the response within the immune compartment of the B cells. The new IFN-β response genes identified by LCL analysis included genes involved in B cell functional pathways, as well as less characterized genes, whose role in B cells merits further investigation. The similarities in gene expression patterns of the response to IFN-β between LCLs and primary B cells highlighted in this study, on the gene level and on the pathways level, support the use of LCLs for discovery and functional analysis of drug effects. For diseases in which B cells have been shown to contribute to pathogenesis, including MS and other autoimmune diseases, studies employing the human derived LCLs, and specifically from patient donors, have an important research potential for comprehension of drug effects on B cells.

## Supporting Information

Table S1 **Primer sequences.**
(DOCX)

Table S2 **Differentially expressed genes up-regulated in response to IFN-β.**
(DOCX)

Table S3 **Top 20 differentially expressed genes down-regulated in response to IFN-β.**
(DOCX)

## References

[1] von Budingen HC, Bar-Or A, Zamvil SS (2011) B cells in multiple sclerosis: connecting the dots. Curr Opin Immunol 23: 713–720. 21983151 10.1016/j.coi.2011.09.003 PMC4188435

[2] Disanto G, Morahan JM, Barnett MH, Giovannoni G, Ramagopalan SV (2012) The evidence for a role of B cells in multiple sclerosis. Neurology 78: 823–832. 22411958 10.1212/WNL.0b013e318249f6f0 PMC3304944

[3] Krumbholz M, Derfuss T, Hohlfeld R, Meinl E (2012) B cells and antibodies in multiple sclerosis pathogenesis and therapy. Nat Rev Neurol 8: 613–623. 23045237 10.1038/nrneurol.2012.203

[4] Casiraghi C, Shanina I, Cho S, Freeman ML, Blackman MA, et al (2012) Gammaherpesvirus latency accentuates EAE pathogenesis: relevance to Epstein-Barr virus and multiple sclerosis. PLoS Pathog 8: e1002715. 22615572 10.1371/journal.ppat.1002715 PMC3355105

[5] Serafini B, Severa M, Columba-Cabezas S, Rosicarelli B, Veroni C, et al (2010) Epstein-Barr virus latent infection and BAFF expression in B cells in the multiple sclerosis brain: implications for viral persistence and intrathecal B-cell activation. J Neuropathol Exp Neurol 69: 677–693. 20535037 10.1097/NEN.0b013e3181e332ec

[6] Hawker K, O'Connor P, Freedman MS, Calabresi PA, Antel J, et al (2009) Rituximab in patients with primary progressive multiple sclerosis: results of a randomized double-blind placebo-controlled multicenter trial. Ann Neurol 66: 460–471. 19847908 10.1002/ana.21867

[7] Hauser SL, Waubant E, Arnold DL, Vollmer T, Antel J, et al (2008) B-cell depletion with rituximab in relapsing-remitting multiple sclerosis. N Engl J Med 358: 676–688. 18272891 10.1056/NEJMoa0706383

[8] Molnarfi N, Schulze-Topphoff U, Weber MS, Patarroyo JC, Prod'homme T, et al (2013) MHC class II-dependent B cell APC function is required for induction of CNS autoimmunity independent of myelin-specific antibodies. J Exp Med 210: 2921–2937. 24323356 10.1084/jem.20130699 PMC3865476

[9] Weber MS, Hemmer B, Cepok S (2011) The role of antibodies in multiple sclerosis. Biochim Biophys Acta 1812: 239–245. 20600871 10.1016/j.bbadis.2010.06.009

[10] Buck D, Hemmer B (2011) Treatment of multiple sclerosis: current concepts and future perspectives. J Neurol 258: 1747–1762. 21637950 10.1007/s00415-011-6101-2

[11] Miller A, Avidan N, Tzunz-Henig N, Glass-Marmor L, Lejbkowicz I, et al (2008) Translation towards personalized medicine in Multiple Sclerosis. J Neurol Sci 274: 68–75. 18789804 10.1016/j.jns.2008.07.028

[12] Hecker M, Paap BK, Goertsches RH, Kandulski O, Fatum C, et al (2011) Reassessment of blood gene expression markers for the prognosis of relapsing-remitting multiple sclerosis. PLoS One 6: e29648. 22216338 10.1371/journal.pone.0029648 PMC3246503

[13] Comabella M, Vandenbroeck K (2011) Pharmacogenomics and multiple sclerosis: moving toward individualized medicine. Curr Neurol Neurosci Rep 11: 484–491. 21701907 10.1007/s11910-011-0211-1

[14] Comabella M, Lunemann JD, Rio J, Sanchez A, Lopez C, et al (2009) A type I interferon signature in monocytes is associated with poor response to interferon-beta in multiple sclerosis. Brain 132: 3353–3365. 19741051 10.1093/brain/awp228

[15] van Boxel-Dezaire AH, Zula JA, Xu Y, Ransohoff RM, Jacobberger JW, et al (2010) Major Differences in the Responses of Primary Human Leukocyte Subsets to IFN-{beta}. J Immunol 185: 5888–5899. 20956346 10.4049/jimmunol.0902314 PMC3244975

[16] Henig N, Avidan N, Mandel I, Staun-Ram E, Ginzburg E, et al (2013) Interferon-beta induces distinct gene expression response patterns in human monocytes versus T cells. PLoS One 8: e62366. 23626809 10.1371/journal.pone.0062366 PMC3633862

[17] Gonzalez-Navajas JM, Lee J, David M, Raz E (2012) Immunomodulatory functions of type I interferons. Nat Rev Immunol 12: 125–135. 22222875 10.1038/nri3133 PMC3727154

[18] Deonarain R, Verma A, Porter AC, Gewert DR, Platanias LC, et al (2003) Critical roles for IFN-beta in lymphoid development, myelopoiesis, and tumor development: links to tumor necrosis factor alpha. Proc Natl Acad Sci U S A 100: 13453–13458. 14597717 10.1073/pnas.2230460100 PMC263835

[19] Green NM, Laws A, Kiefer K, Busconi L, Kim YM, et al (2009) Murine B cell response to TLR7 ligands depends on an IFN-beta feedback loop. J Immunol 183: 1569–1576. 19587008 10.4049/jimmunol.0803899 PMC2929820

[20] Poovassery JS, Bishop GA (2012) Type I IFN receptor and the B cell antigen receptor regulate TLR7 responses via distinct molecular mechanisms. J Immunol 189: 1757–1764. 22786773 10.4049/jimmunol.1200624

[21] Walsh ER, Pisitkun P, Voynova E, Deane JA, Scott BL, et al (2012) Dual signaling by innate and adaptive immune receptors is required for TLR7-induced B-cell-mediated autoimmunity. Proc Natl Acad Sci U S A 109: 16276–16281. 22988104 10.1073/pnas.1209372109 PMC3479588

[22] Krumbholz M, Faber H, Steinmeyer F, Hoffmann LA, Kumpfel T, et al (2008) Interferon-beta increases BAFF levels in multiple sclerosis: implications for B cell autoimmunity. Brain 131: 1455–1463. 18474519 10.1093/brain/awn077

[23] Ramgolam VS, Sha Y, Marcus KL, Choudhary N, Troiani L, et al (2011) B cells as a therapeutic target for IFN-beta in relapsing-remitting multiple sclerosis. J Immunol 186: 4518–4526. 21368231 10.4049/jimmunol.1000271

[24] Wroblewski JM, Copple A, Batson LP, Landers CD, Yannelli JR (2002) Cell surface phenotyping and cytokine production of Epstein-Barr Virus (EBV)-transformed lymphoblastoid cell lines (LCLs). J Immunol Methods 264: 19–28. 12191505 10.1016/s0022-1759(01)00565-8

[25] Sie L, Loong S, Tan EK (2009) Utility of lymphoblastoid cell lines. J Neurosci Res 87: 1953–1959. 19224581 10.1002/jnr.22000

[26] Caliskan M, Cusanovich DA, Ober C, Gilad Y (2011) The effects of EBV transformation on gene expression levels and methylation profiles. Hum Mol Genet 20: 1643–1652. 21289059 10.1093/hmg/ddr041 PMC3063990

[27] Cheung VG, Conlin LK, Weber TM, Arcaro M, Jen KY, et al (2003) Natural variation in human gene expression assessed in lymphoblastoid cells. Nat Genet 33: 422–425. 12567189 10.1038/ng1094

[28] Hu VW, Frank BC, Heine S, Lee NH, Quackenbush J (2006) Gene expression profiling of lymphoblastoid cell lines from monozygotic twins discordant in severity of autism reveals differential regulation of neurologically relevant genes. BMC Genomics 7: 118. 16709250 10.1186/1471-2164-7-118 PMC1525191

[29] Welsh M, Mangravite L, Medina MW, Tantisira K, Zhang W, et al (2009) Pharmacogenomic discovery using cell-based models. Pharmacol Rev 61: 413–429. 20038569 10.1124/pr.109.001461 PMC2802425

[30] Morag A, Kirchheiner J, Rehavi M, Gurwitz D (2010) Human lymphoblastoid cell line panels: novel tools for assessing shared drug pathways. Pharmacogenomics 11: 327–340. 20235789 10.2217/pgs.10.27

[31] Disanto G, Sandve GK, Berlanga-Taylor AJ, Morahan JM, Dobson R, et al (2012) Genomic regions associated with multiple sclerosis are active in B cells. PLoS One 7: e32281. 22396755 10.1371/journal.pone.0032281 PMC3292555

[32] Disanto G, Sandve GK, Berlanga-Taylor AJ, Ragnedda G, Morahan JM, et al (2012) Vitamin D receptor binding, chromatin states and association with multiple sclerosis. Hum Mol Genet 21: 3575–3586. 22595971 10.1093/hmg/dds189 PMC3406756

[33] McDonald WI, Compston A, Edan G, Goodkin D, Hartung HP, et al (2001) Recommended diagnostic criteria for multiple sclerosis: guidelines from the International Panel on the diagnosis of multiple sclerosis. Ann Neurol 50: 121–127. 11456302 10.1002/ana.1032

[34] Poser CM, Paty DW, Scheinberg L, McDonald WI, Davis FA, et al (1983) New diagnostic criteria for multiple sclerosis: guidelines for research protocols. Ann Neurol 13: 227–231. 6847134 10.1002/ana.410130302

[35] Lee JE, Nam HY, Shim SM, Bae GR, Han BG, et al (2010) Expression phenotype changes of EBV-transformed lymphoblastoid cell lines during long-term subculture and its clinical significance. Cell Prolif 43: 378–384. 20590663 10.1111/j.1365-2184.2010.00687.x PMC6496229

[36] Buchwalder PA, Buclin T, Trinchard I, Munafo A, Biollaz J (2000) Pharmacokinetics and pharmacodynamics of IFN-beta 1a in healthy volunteers. J Interferon Cytokine Res 20: 857–866. 11054273 10.1089/10799900050163226

[37] Khan OA, Dhib-Jalbut SS (1998) Serum interferon beta-1a (Avonex) levels following intramuscular injection in relapsing-remitting MS patients. Neurology 51: 738–742. 9748019 10.1212/wnl.51.3.738

[38] Mandel I, Paperna T, Glass-Marmor L, Volkowich A, Badarny S, et al (2012) Tight junction proteins expression and modulation in immune cells and multiple sclerosis. J Cell Mol Med 16: 765–775. 21762372 10.1111/j.1582-4934.2011.01380.x PMC3822847

[39] Livak KJ, Schmittgen TD (2001) Analysis of relative gene expression data using real-time quantitative PCR and the 2(-Delta Delta C(T)) Method. Methods 25: 402–408. 11846609 10.1006/meth.2001.1262

[40] Benjamini Y, Hochberg Y (1995) Controlling the false discovery rate: a practical and powerful approach to multiple testing. Roy Stat Soc B 57: 289–300.

[41] Rusinova I, Forster S, Yu S, Kannan A, Masse M, et al (2013) Interferome v2.0: an updated database of annotated interferon-regulated genes. Nucleic Acids Res 41: D1040–1046. 23203888 10.1093/nar/gks1215 PMC3531205

[42] Dorner M, Zucol F, Berger C, Byland R, Melroe GT, et al (2008) Distinct ex vivo susceptibility of B-cell subsets to epstein-barr virus infection according to differentiation status and tissue origin. J Virol 82: 4400–4412. 18321980 10.1128/JVI.02630-07 PMC2293034

[43] Monson NL (2008) The natural history of B cells. Curr Opin Neurol 21 Suppl 1S3–8. 18388800 10.1097/01.wco.0000313358.53553.c7

[44] Ohtsuka T, Nakanishi H, Ikeda W, Satoh A, Momose Y, et al (1998) Nexilin: a novel actin filament-binding protein localized at cell-matrix adherens junction. J Cell Biol 143: 1227–1238. 9832551 10.1083/jcb.143.5.1227 PMC2133087

[45] Spicer AP, Joo A, Bowling RA Jr (2003) A hyaluronan binding link protein gene family whose members are physically linked adjacent to chondroitin sulfate proteoglycan core protein genes: the missing links. J Biol Chem 278: 21083–21091. 12663660 10.1074/jbc.M213100200

[46] Miyashita M, Oshiumi H, Matsumoto M, Seya T (2011) DDX60, a DEXD/H box helicase, is a novel antiviral factor promoting RIG-I-like receptor-mediated signaling. Mol Cell Biol 31: 3802–3819. 21791617 10.1128/MCB.01368-10 PMC3165724

[47] Schoggins JW, Wilson SJ, Panis M, Murphy MY, Jones CT, et al (2011) A diverse range of gene products are effectors of the type I interferon antiviral response. Nature 472: 481–485. 21478870 10.1038/nature09907 PMC3409588

[48] Durai R, Davies M, Yang W, Yang SY, Seifalian A, et al (2006) Biology of insulin-like growth factor binding protein-4 and its role in cancer (review). Int J Oncol 28: 1317–1325. 16685432

[49] Kisielow M, Kisielow J, Capoferri-Sollami G, Karjalainen K (2005) Expression of lymphocyte activation gene 3 (LAG-3) on B cells is induced by T cells. Eur J Immunol 35: 2081–2088. 15971272 10.1002/eji.200526090

[50] Sierro S, Romero P, Speiser DE (2011) The CD4-like molecule LAG-3, biology and therapeutic applications. Expert Opin Ther Targets 15: 91–101. 21142803 10.1517/14712598.2011.540563

[51] Batten M, Ramamoorthi N, Kljavin NM, Ma CS, Cox JH, et al (2010) IL-27 supports germinal center function by enhancing IL-21 production and the function of T follicular helper cells. J Exp Med 207: 2895–2906. 21098093 10.1084/jem.20100064 PMC3005229

[52] Harker JA, Dolgoter A, Zuniga EI (2013) Cell-intrinsic IL-27 and gp130 cytokine receptor signaling regulates virus-specific CD4(+) T cell responses and viral control during chronic infection. Immunity 39: 548–559. 23993651 10.1016/j.immuni.2013.08.010 PMC4701058

[53] Benschop RJ, Cambier JC (1999) B cell development: signal transduction by antigen receptors and their surrogates. Curr Opin Immunol 11: 143–151. 10322153 10.1016/s0952-7915(99)80025-9

[54] Chesik D, De Keyser J, Glazenburg L, Wilczak N (2006) Insulin-like growth factor binding proteins: regulation in chronic active plaques in multiple sclerosis and functional analysis of glial cells. Eur J Neurosci 24: 1645–1652. 17004928 10.1111/j.1460-9568.2006.05034.x

[55] Zhou R, Flaswinkel H, Schneider MR, Lahm H, Hoeflich A, et al (2004) Insulin-like growth factor-binding protein-4 inhibits growth of the thymus in transgenic mice. J Mol Endocrinol 32: 349–364. 15072544 10.1677/jme.0.0320349

[56] Winkelmann R, Sandrock L, Porstner M, Roth E, Mathews M, et al (2011) B cell homeostasis and plasma cell homing controlled by Kruppel-like factor 2. Proc Natl Acad Sci U S A 108: 710–715. 21187409 10.1073/pnas.1012858108 PMC3021026

[57] Workman CJ, Vignali DA (2005) Negative regulation of T cell homeostasis by lymphocyte activation gene-3 (CD223). J Immunol 174: 688–695. 15634887 10.4049/jimmunol.174.2.688

[58] Okazaki T, Okazaki IM, Wang J, Sugiura D, Nakaki F, et al (2011) PD-1 and LAG-3 inhibitory co-receptors act synergistically to prevent autoimmunity in mice. J Exp Med 208: 395–407. 21300912 10.1084/jem.20100466 PMC3039848

[59] von Budingen HC, Kuo TC, Sirota M, van Belle CJ, Apeltsin L, et al (2012) B cell exchange across the blood-brain barrier in multiple sclerosis. J Clin Invest 122: 4533–4543. 23160197 10.1172/JCI63842 PMC3533544

[60] Yoshimoto T, Okada K, Morishima N, Kamiya S, Owaki T, et al (2004) Induction of IgG2a class switching in B cells by IL-27. J Immunol 173: 2479–2485. 15294962 10.4049/jimmunol.173.4.2479

[61] Lappalainen T, Sammeth M, Friedlander MR, t Hoen PA, Monlong J, et al (2013) Transcriptome and genome sequencing uncovers functional variation in humans. Nature 501: 506–511. 24037378 10.1038/nature12531 PMC3918453

[62] Min JL, Taylor JM, Richards JB, Watts T, Pettersson FH, et al (2011) The use of genome-wide eQTL associations in lymphoblastoid cell lines to identify novel genetic pathways involved in complex traits. PLoS One 6: e22070. 21789213 10.1371/journal.pone.0022070 PMC3137612

[63] Bolotin E, Armendariz A, Kim K, Heo SJ, Boffelli D, et al (2014) Statin-induced changes in gene expression in EBV-transformed and native B-cells. Hum Mol Genet 23: 1202–1210. 24179175 10.1093/hmg/ddt512 PMC3919007

[64] Sanders AR, Goring HH, Duan J, Drigalenko EI, Moy W, et al (2013) Transcriptome study of differential expression in schizophrenia. Hum Mol Genet 22: 5001–5014. 23904455 10.1093/hmg/ddt350 PMC3836479

[65] Squassina A, Costa M, Congiu D, Manchia M, Angius A, et al (2013) Insulin-like growth factor 1 (IGF-1) expression is up-regulated in lymphoblastoid cell lines of lithium responsive bipolar disorder patients. Pharmacol Res 73: 1–7. 23619527 10.1016/j.phrs.2013.04.004

[66] Morag A, Pasmanik-Chor M, Oron-Karni V, Rehavi M, Stingl JC, et al (2011) Genome-wide expression profiling of human lymphoblastoid cell lines identifies CHL1 as a putative SSRI antidepressant response biomarker. Pharmacogenomics 12: 171–184. 21332311 10.2217/pgs.10.185

[67] Morag A, Oved K, Gurwitz D (2013) Sex differences in human lymphoblastoid cells sensitivities to antipsychotic drugs. J Mol Neurosci 49: 554–558. 22760742 10.1007/s12031-012-9852-z

[68] Mestas J, Hughes CC (2004) Of mice and not men: differences between mouse and human immunology. J Immunol 172: 2731–2738. 14978070 10.4049/jimmunol.172.5.2731

[69] Pieper K, Grimbacher B, Eibel H (2013) B-cell biology and development. J Allergy Clin Immunol 131: 959–971. 23465663 10.1016/j.jaci.2013.01.046

[70] Rogge L, D'Ambrosio D, Biffi M, Penna G, Minetti LJ, et al (1998) The role of Stat4 in species-specific regulation of Th cell development by type I IFNs. J Immunol 161: 6567–6574. 9862683

[71] Abramovich C, Chebath J, Revel M (1994) The human interferon alpha-receptor protein confers differential responses to human interferon-beta versus interferon-alpha subtypes in mouse and hamster cell transfectants. Cytokine 6: 414–424. 7948750 10.1016/1043-4666(94)90066-3

[72] Croze E, Yamaguchi KD, Knappertz V, Reder AT, Salamon H (2013) Interferon-beta-1b-induced short- and long-term signatures of treatment activity in multiple sclerosis. Pharmacogenomics J 13: 443–451. 22711062 10.1038/tpj.2012.27 PMC3793239

[73] Fowler T, Sen R, Roy AL (2011) Regulation of primary response genes. Mol Cell 44: 348–360. 22055182 10.1016/j.molcel.2011.09.014 PMC3212756

[74] Pone EJ, Zan H, Zhang J, Al-Qahtani A, Xu Z, et al (2010) Toll-like receptors and B-cell receptors synergize to induce immunoglobulin class-switch DNA recombination: relevance to microbial antibody responses. Crit Rev Immunol 30: 1–29. 20370617 10.1615/critrevimmunol.v30.i1.10 PMC3038989

[75] McEwan WA, Tam JC, Watkinson RE, Bidgood SR, Mallery DL, et al (2013) Intracellular antibody-bound pathogens stimulate immune signaling via the Fc receptor TRIM21. Nat Immunol 14: 327–336. 23455675 10.1038/ni.2548 PMC3672961

[76] Pertel T, Hausmann S, Morger D, Zuger S, Guerra J, et al (2011) TRIM5 is an innate immune sensor for the retrovirus capsid lattice. Nature 472: 361–365. 21512573 10.1038/nature09976 PMC3081621

[77] Versteeg GA, Rajsbaum R, Sanchez-Aparicio MT, Maestre AM, Valdiviezo J, et al (2013) The E3-ligase TRIM family of proteins regulates signaling pathways triggered by innate immune pattern-recognition receptors. Immunity 38: 384–398. 23438823 10.1016/j.immuni.2012.11.013 PMC3584420

