# Supplementary material for: Gene Expression Profiling of the Response to Interferon Beta in Epstein-Barr-Transformed and Primary B Cells of Patients with Multiple Sclerosis
Source: PLoS One. 2014 Jul 15;9(7):e102331. doi: 10.1371/journal.pone.0102331 (PMC4099420; doi:10.1371/journal.pone.0102331)
Supplement: Table S1 — Primer sequences. (DOCX) [file pone.0102331.s001.docx]

| Table S1. Primer sequences | | |
| --- | --- | --- |
| Gene Symbol | Forward | Reverse |
| TNFSF13A/BAFF | CGTTCAGGGTCCAGAAGAAA | AAGCTGAGAAGCCATGGAAC |
| CD79B | GCGAGTCATGGGATTCAGCACC | TGCCAGCCTTGCTGTCATCC |
| DDX60L | TGGGATGCGTATGGGACAG | TCAAGGAGTCACTGATAGCC |
| HAPLN3 | GGCTTCTACTACTCCAACAG | GGTAGGTGAACAGGGTCTC |
| IGFBP4 | CCACGAGGACCTCTACATC | GTCCACACACCAGCACTTG |
| IL27RA^a^ | CCGAGTTACACCTCCAGAGC | AGACATGGTGAGCTGTTCCC |
| KLF2^a^ | TTGCAGTGGTAGGGCTTCTC | ACTCACACCTGCAGCTACGC |
| LAG-3^a^ | TCACATTGGCAATCATCACA | CACAAAGCGTTCTTGTCCAG |
| NEXN | CGACGAACAGAGGAGGAAC | TTCTCTGCTCAATCCAAAGGT |
| SYK^b^ | GTGTCATTCAATCCGTATGAGCC | TTTCGGTCCAGGTAAACCTCC |
| UBE2D2 | ATTGAATGATCTGGCACGGG | TGTCATTTGGCCCCATTATTG |
| a. qPrimerDepot, b. PrimerBank. Other primers were designed by us. | | |
