# Supplementary material for: Gene Expression Profiling of the Response to Interferon Beta in Epstein-Barr-Transformed and Primary B Cells of Patients with Multiple Sclerosis
Source: PLoS One. 2014 Jul 15;9(7):e102331. doi: 10.1371/journal.pone.0102331 (PMC4099420; doi:10.1371/journal.pone.0102331)
Supplement: Table S2 — Differentially expressed genes up-regulated in response to IFN-β. (DOCX) [file pone.0102331.s002.docx]

| Table S2. Differentially expressed genes up-regulated in response to IFN-β ^a^. | | | |
| --- | --- | --- | --- |
| **Gene Symbol^b^** | **Definition** | **Fold Change** | **Adjusted p-Value** |
| RSAD2 | radical S-adenosyl methionine domain containing 2 (RSAD2). | 12.2 | 8.4E-24 |
| OASL | 2'-5'-oligoadenylate synthetase-like (OASL), transcript variant 2. | 8.8 | 1.3E-22 |
| USP18 | ubiquitin specific peptidase 18 (USP18). | 7.8 | 3.5E-21 |
| TNFSF10 | tumor necrosis factor (ligand) superfamily, member 10 (TNFSF10). | 11.6 | 5.5E-21 |
| EPSTI1 | epithelial stromal interaction 1 (breast) (EPSTI1), transcript variant 2. | 4.7 | 1.6E-20 |
| IFIT1 | interferon-induced protein with tetratricopeptide repeats 1 (IFIT1), transcript variant 2. | 16.3 | 1.6E-20 |
| IFIT3 | interferon-induced protein with tetratricopeptide repeats 3 (IFIT3). | 9.0 | 2.9E-20 |
| IRF7 | interferon regulatory factor 7 (IRF7), transcript variant b. | 4.6 | 2.9E-20 |
| ISG15 | ISG15 ubiquitin-like modifier (ISG15). | 5.1 | 2.9E-20 |
| **CMPK2** | cytidine monophosphate (UMP-CMP) kinase 2, mitochondrial (CMPK2), nuclear gene encoding mitochondrial protein. | 4.5 | 3.8E-20 |
| NT5C3 | 5'-nucleotidase, cytosolic III (NT5C3), transcript variant 1. | 4.6 | 1.1E-19 |
| **USP41** | PREDICTED: ubiquitin specific peptidase 41 (USP41). | 3.8 | 1.3E-19 |
| TLR7 | toll-like receptor 7 (TLR7). | 3.4 | 1.7E-19 |
| HERC6 | hect domain and RLD 6 (HERC6), transcript variant 1. | 4.7 | 5.2E-19 |
| SP110 | SP110 nuclear body protein (SP110), transcript variant b. | 3.7 | 5.2E-19 |
| IFIT2 | interferon-induced protein with tetratricopeptide repeats 2 (IFIT2). | 7.6 | 7.2E-19 |
| DHX58 | DEXH (Asp-Glu-X-His) box polypeptide 58 (DHX58). | 3.7 | 7.9E-19 |
| HERC5 | hect domain and RLD 5 (HERC5). | 5.1 | 8.7E-19 |
| PLSCR1 | phospholipid scramblase 1 (PLSCR1). | 4.1 | 1.3E-18 |
| DDX58 | DEAD (Asp-Glu-Ala-Asp) box polypeptide 58 (DDX58). | 3.6 | 4.2E-18 |
| PARP14 | poly (ADP-ribose) polymerase family, member 14 (PARP14). | 3.1 | 4.2E-18 |
| **PRIC285** | peroxisomal proliferator-activated receptor A interacting complex 285 (PRIC285), transcript variant 2. | 5.8 | 4.9E-18 |
| **SAMD9L** | sterile alpha motif domain containing 9-like (SAMD9L). | 5.8 | 1.4E-17 |
| STAT2 | signal transducer and activator of transcription 2, 113kDa (STAT2). | 3.1 | 1.8E-17 |
| IFI35 | interferon-induced protein 35 (IFI35). | 3.4 | 3.3E-17 |
| PARP9 | poly (ADP-ribose) polymerase family, member 9 (PARP9). | 4.0 | 3.4E-17 |
| SAMD9 | sterile alpha motif domain containing 9 (SAMD9). | 4.1 | 3.4E-17 |
| EIF2AK2 | eukaryotic translation initiation factor 2-alpha kinase 2 (EIF2AK2). | 2.8 | 1.1E-16 |
| **TNFSF13B** | tumor necrosis factor (ligand) superfamily, member 13b (TNFSF13B), transcript variant 1. | 4.3 | 1.3E-16 |
| IFIH1 | interferon induced with helicase C domain 1 (IFIH1). | 3.3 | 1.6E-16 |
| ISG20 | interferon stimulated exonuclease gene 20kDa (ISG20). | 3.1 | 2.9E-16 |
| **LBA1** | lupus brain antigen 1 (LBA1). | 4.2 | 3.0E-16 |
| **DDX60L** | DEAD (Asp-Glu-Ala-Asp) box polypeptide 60-like (DDX60L). | 4.1 | 4.4E-16 |
| OAS2 | 2'-5'-oligoadenylate synthetase 2, 69/71kDa (OAS2), transcript variant 3. | 4.4 | 4.4E-16 |
| MX2 | myxovirus (influenza virus) resistance 2 (mouse) (MX2). | 5.2 | 6.2E-16 |
| OAS3 | 2'-5'-oligoadenylate synthetase 3, 100kDa (OAS3). | 4.0 | 1.4E-15 |
| **PARP10** | poly (ADP-ribose) polymerase family, member 10 (PARP10). | 2.8 | 1.7E-15 |
| **C14ORF149** | chromosome 14 open reading frame 149 (C14orf149). | 3.1 | 2.0E-15 |
| DDX60 | DEAD (Asp-Glu-Ala-Asp) box polypeptide 60 (DDX60). | 3.9 | 2.4E-15 |
| UBE2L6 | ubiquitin-conjugating enzyme E2L 6 (UBE2L6), transcript variant 1. | 2.4 | 2.5E-15 |
| **XRN1** | 5'-3' exoribonuclease 1 (XRN1). | 2.7 | 3.9E-15 |
| STAT1 | signal transducer and activator of transcription 1, 91kDa (STAT1), transcript variant alpha. | 3.0 | 3.9E-15 |
| XAF1 | XIAP associated factor 1 (XAF1), transcript variant 2. | 4.5 | 4.6E-15 |
| SP100 | SP100 nuclear antigen (SP100), transcript variant 1. | 2.3 | 5.1E-15 |
| IFI44 | interferon-induced protein 44 (IFI44). | 3.3 | 5.3E-15 |
| FBXO6 | F-box protein 6 (FBXO6). | 3.3 | 8.4E-15 |
| CHMP5 | chromatin modifying protein 5 (CHMP5). | 2.5 | 1.0E-14 |
| OAS1 | 2',5'-oligoadenylate synthetase 1, 40/46kDa (OAS1), transcript variant 3. | 3.6 | 1.1E-14 |
| MYD88 | myeloid differentiation primary response gene (88) (MYD88). | 2.7 | 1.3E-14 |
| C19ORF66 | chromosome 19 open reading frame 66 (C19orf66). | 2.9 | 1.6E-14 |
| PARP12 | poly (ADP-ribose) polymerase family, member 12 (PARP12). | 3.3 | 1.8E-14 |
| IFI16 | interferon, gamma-inducible protein 16 (IFI16). | 2.9 | 2.2E-14 |
| TRIM22 | tripartite motif-containing 22 (TRIM22). | 2.7 | 2.5E-14 |
| TRIM21 | tripartite motif-containing 21 (TRIM21). | 2.4 | 2.9E-14 |
| TDRD7 | tudor domain containing 7 (TDRD7). | 2.5 | 7.1E-14 |
| **MNDA** | myeloid cell nuclear differentiation antigen (MNDA). | 3.6 | 1.0E-13 |
| APOL3 | apolipoprotein L, 3 (APOL3), transcript variant beta/a. | 2.8 | 1.2E-13 |
| PPM1K | protein phosphatase 1K (PP2C domain containing) (PPM1K). | 2.8 | 1.2E-13 |
| IFI27 | interferon, alpha-inducible protein 27 (IFI27), transcript variant 2. | 3.2 | 1.2E-13 |
| **HRASLS2** | HRAS-like suppressor 2 (HRASLS2). | 2.4 | 3.6E-13 |
| **LOC400759** | similar to Interferon-induced guanylate-binding protein 1 (GTP-binding protein 1) (Guanine nucleotide-binding protein 1) (HuGBP-1) (LOC400759) on chromosome 1. | 2.3 | 3.7E-13 |
| **DOPEY1** | dopey family member 1 (DOPEY1). | 2.3 | 3.7E-13 |
| **LOC728216** | PREDICTED: similar to ubiquitin specific peptidase 18 (LOC728216). | 2.1 | 4.0E-13 |
| GBP1 | guanylate binding protein 1, interferon-inducible, 67kDa (GBP1). | 3.5 | 4.3E-13 |
| **EXOSC9** | exosome component 9 (EXOSC9), transcript variant 1. | 2.2 | 4.6E-13 |
| **KIAA1618** | KIAA1618 (KIAA1618). | 2.8 | 4.7E-13 |
| NUB1 | negative regulator of ubiquitin-like proteins 1 (NUB1). | 2.4 | 4.7E-13 |
| IFITM1 | interferon induced transmembrane protein 1 (9-27) (IFITM1). | 2.1 | 5.3E-13 |
| **ENDOD1** | endonuclease domain containing 1 (ENDOD1). | 2.9 | 5.4E-13 |
| **HAPLN3** | hyaluronan and proteoglycan link protein 3 (HAPLN3). | 2.7 | 6.6E-13 |
| **PI4K2B** | phosphatidylinositol 4-kinase type 2 beta (PI4K2B). | 2.4 | 8.5E-13 |
| ADAR | adenosine deaminase, RNA-specific (ADAR), transcript variant 2. | 2.1 | 2.3E-12 |
| **LOC389386** | PREDICTED: misc_RNA (LOC389386), partial miscRNA. | 2.1 | 4.4E-12 |
| RTP4 | receptor (chemosensory) transporter protein 4 (RTP4). | 2.7 | 4.5E-12 |
| ZNFX1 | zinc finger, NFX1-type containing 1 (ZNFX1). | 2.4 | 5.3E-12 |
| GMPR | guanosine monophosphate reductase (GMPR). | 2.6 | 6.4E-12 |
| **IGFBP4** | insulin-like growth factor binding protein 4 (IGFBP4). | 3.5 | 6.5E-12 |
| TRIM25 | tripartite motif-containing 25 (TRIM25). | 2.2 | 9.3E-12 |
| LAG3 | lymphocyte-activation gene 3 (LAG3). | 4.2 | 9.5E-12 |
| IFI44L | interferon-induced protein 44-like (IFI44L). | 3.0 | 1.0E-11 |
| CD38 | CD38 molecule (CD38). | 3.2 | 1.2E-11 |
| **NEXN** | nexilin (F actin binding protein) (NEXN). | 3.5 | 1.5E-11 |
| GBP4 | guanylate binding protein 4 (GBP4). | 3.4 | 1.6E-11 |
| **LOC643384** | PREDICTED: hypothetical LOC643384 (LOC643384). | 2.7 | 2.0E-11 |
| IFI6 | interferon, alpha-inducible protein 6 (IFI6), transcript variant 3. | 2.9 | 2.2E-11 |
| **DTX3L** | deltex 3-like (Drosophila) (DTX3L). | 2.2 | 2.6E-11 |
| GCH1 | GTP cyclohydrolase 1 (GCH1), transcript variant 4. | 2.2 | 3.6E-11 |
| **UNC93B1** | unc-93 homolog B1 (C. elegans) (UNC93B1). | 2.3 | 3.6E-11 |
| WARS | tryptophanyl-tRNA synthetase (WARS), transcript variant 2. | 2.8 | 3.6E-11 |
| **DYNLT1** | dynein, light chain, Tctex-type 1 (DYNLT1). | 2.2 | 3.9E-11 |
| UBA7 | ubiquitin-like modifier activating enzyme 7 (UBA7). | 2.1 | 4.7E-11 |
| **PRKAG2** | protein kinase, AMP-activated, gamma 2 non-catalytic subunit (PRKAG2), transcript variant b. | 2.1 | 5.0E-11 |
| **EHD4** | EH-domain containing 4 (EHD4). | 2.0 | 8.6E-11 |
| **HSH2D** | hematopoietic SH2 domain containing (HSH2D). | 2.0 | 1.2E-10 |
| **BRDG1** | BCR downstream signaling 1 (BRDG1). | 2.2 | 1.3E-10 |
| **HS.125087** | AGENCOURT_7914287 NIH_MGC_71 cDNA clone IMAGE:6156595 5 sequence | 2.6 | 3.8E-10 |
| TAP1 | transporter 1, ATP-binding cassette, sub-family B (MDR/TAP) (TAP1). | 2.0 | 6.8E-10 |
| **PDGFRL** | platelet-derived growth factor receptor-like (PDGFRL). | 2.5 | 5.1E-09 |
| INPP1 | inositol polyphosphate-1-phosphatase (INPP1). | 2.1 | 8.0E-09 |
| **STAP1** | signal transducing adaptor family member 1 (STAP1). | 2.3 | 8.6E-09 |
| AIM2 | absent in melanoma 2 (AIM2). | 2.2 | 1.2E-08 |
| LAMP3 | lysosomal-associated membrane protein 3 (LAMP3). | 2.3 | 1.4E-08 |
| GBP5 | guanylate binding protein 5 (GBP5). | 2.8 | 7.9E-08 |
| **ARHGEF3** | Rho guanine nucleotide exchange factor (GEF) 3 (ARHGEF3). | 2.5 | 1.2E-07 |
| **HESX1** | HESX homeobox 1 (HESX1). | 2.1 | 1.6E-07 |
| WDFY1 | WD repeat and FYVE domain containing 1 (WDFY1). | 2.0 | 4.6E-07 |
| **CCL3L1** | chemokine (C-C motif) ligand 3-like 1 (CCL3L1). | 2.6 | 7.6E-07 |
| IFITM3 | interferon induced transmembrane protein 3 (1-8U) (IFITM3). | 3.7 | 1.0E-06 |
| **ZBP1** | Z-DNA binding protein 1 (ZBP1). | 2.5 | 3.3E-06 |
| **HES4** | hairy and enhancer of split 4 (Drosophila) (HES4). | 2.2 | 6.0E-06 |
| KCTD14 | potassium channel tetramerisation domain containing 14 (KCTD14). | 2.1 | 9.7E-06 |
| **ENPP2** | ectonucleotide pyrophosphatase/phosphodiesterase 2 (ENPP2), transcript variant 2. | 2.5 | 1.3E-05 |
| CXCL10 | chemokine (C-X-C motif) ligand 10 (CXCL10). | 4.3 | 4.0E-05 |
| **SLFN5** | schlafen family member 5 (SLFN5). | 2.1 | 1.8E-04 |
| **SIGLEC14** | sialic acid binding Ig-like lectin 14 (SIGLEC14). | 2.0 | 4.7E-04 |

^a^ One way ANOVA analysis, genes with fold change ≥ 2, and FDR adjusted P-value ≤ 0.05.

^b^ Genes that are not up-regulated in LCLs in comparison to B cells, based on data from Caliskan et al., are marked in bold.

Caliskan, M., Cusanovich, D.A., Ober, C. and Gilad, Y. (2011) The effects of EBV transformation on gene expression levels and methylation profiles. *Hum. Mol. Genet.*, **20**, 1643-1652.
