# Supplementary material for: Gene Expression Profiling of the Response to Interferon Beta in Epstein-Barr-Transformed and Primary B Cells of Patients with Multiple Sclerosis
Source: PLoS One. 2014 Jul 15;9(7):e102331. doi: 10.1371/journal.pone.0102331 (PMC4099420; doi:10.1371/journal.pone.0102331)
Supplement: Table S3 — Top 20 differentially expressed genes down-regulated in response to IFN-β. (DOCX) [file pone.0102331.s003.docx]

| Table S3. Top 20 differentially expressed genes down-regulated in response to IFN-β ^a^. | | | |
| --- | --- | --- | --- |
| **Gene Symbol** | **Definition** | **Fold Change** | **Adjusted p-Value** |
| KLF2 | Kruppel-like factor 2 (lung) (KLF2), mRNA. | 0.52 | 0.0004 |
| CERK | ceramide kinase (CERK), transcript variant 1, mRNA. | 0.52 | 3E-10 |
| TNS3 | tensin 3 (TNS3), mRNA. | 0.52 | 2E-05 |
| C13ORF15 | chromosome 13 open reading frame 15 (C13orf15), mRNA. | 0.54 | 0.0001 |
| MGC87042 | PREDICTED: similar to Six transmembrane epithelial antigen of prostate (MGC87042), mRNA. | 0.55 | 0.016 |
| LAMC1 | laminin, gamma 1 (formerly LAMB2) (LAMC1), mRNA. | 0.56 | 1E-05 |
| SGK | serum/glucocorticoid regulated kinase (SGK), mRNA. | 0.57 | 0.0003 |
| GLS | glutaminase (GLS), mRNA. | 0.57 | 5E-09 |
| TOX2 | TOX high mobility group box family member 2 (TOX2), transcript variant 1, mRNA. | 0.58 | 0.0001 |
| IL27RA | interleukin 27 receptor, alpha (IL27RA), mRNA. | 0.58 | 4E-07 |
| TP53INP1 | tumor protein p53 inducible nuclear protein 1 (TP53INP1), mRNA. | 0.58 | 1E-06 |
| C20ORF100 | chromosome 20 open reading frame 100 (C20orf100), mRNA. | 0.59 | 0.0005 |
| PRAGMIN | homolog of rat pragma of Rnd2 (PRAGMIN), mRNA. | 0.6 | 0.0007 |
| STEAP1 | six transmembrane epithelial antigen of the prostate 1 (STEAP1), mRNA. | 0.61 | 0.0143 |
| RASSF2 | Ras association (RalGDS/AF-6) domain family 2 (RASSF2), transcript variant 2, mRNA. | 0.61 | 2E-05 |
| ATXN1 | ataxin 1 (ATXN1), mRNA. | 0.61 | 7E-08 |
| LRMP | lymphoid-restricted membrane protein (LRMP), mRNA. | 0.61 | 0.0003 |
| SLC25A42 | solute carrier family 25, member 42 (SLC25A42), mRNA. | 0.61 | 3E-07 |
| OXTR | oxytocin receptor (OXTR), mRNA. | 0.62 | 0.0015 |
| CBLB | Cas-Br-M (murine) ecotropic retroviral transforming sequence b (CBLB), mRNA. | 0.62 | 0.0002 |

^a^ One way ANOVA analysis, genes top-listed by fold change, with FDR adjusted P-value ≤ 0.05. Genes included in this table all have a fold change of less than two, and are not included in the Venn diagram shown in Figure 3B.
